# Elements of Medical Jurisprudence

**Published:** 1860-05

**Authors:** 


ART. V.—*Elements of Medical Jurisprudence.* By THEODRIC ROMEYN BECK, M.D., LL.D., etc., etc., and JOHN B. BECK, M.D., etc., etc. Eleventh Edition. With Notes by an Association of the Friends of Drs. Beck. The whole revised by C. R. GILMAN, M.D., Professor of Medical Jurisprudence in the College of Physicians and Surgeons of New York. In Two Volumes. pp. 884 and 1003. Philadelphia: J. B. Lippincott & Co., 1860.

THE appearance of a new edition of the world-renowned work on medical jurisprudence of the brothers Beck, gives rise to emotions of a grave as well as of an inspiring nature. One of the original authors, Dr. John B. Beck, has been dead some years; the other, Dr. Theodric Romeyn, by whose plastic skill the work gained form, and shape, and distinctive features, and who continued to watch and nourish it by regular supplies, has also more recently paid the great debt, and can no longer enjoy the fame which he had so well earned by his labors. But friendship has evoked intelligent, sympathizing spirits to continue in the course marked out by those who have gone before them, and thus, by "a labor of love and respect," the world is favored with a new and enlarged edition of Beck's Elements of Medical Jurisprudence.

We learn from a short and modest preface by Dr. Gilman, that the task of preparing a new edition for the press was confided to him by the family of Dr. T. R. Beck; and at the same time a large amount of matter was placed at his disposal, which the author had, with characteristic industry collected for a new edition of his treatise on Medical Jurisprudence. Dr. Gilman, not relying on his own ability to do entire justice to such a trust, sought aid from the friends of Dr. Beck. The appeal was promptly responded to, and he was enabled to place most of the more important chapters in competent hands. The list of these his collaborators is given. Taken in the order of contributions furnished by them it consists of Dr. R. H. Coolidge, U. S. A., on Feigned Diseases; Dr. Austin Flint, on Impotence and Sterility, Doubtful Sex and Rape; Dr. J. P. White, on Pregnancy; Dr. Tilden Brown, on Mental Alienation; Dr. John Watson, Wounds on the Living Body; Dr. B. W. McCready, on Poisons; Dr. Samuel St. John, on Irritant and Narcotic Poisons; and Dr. C. R. Gilman, on Medical Evidence, and Insurance upon Lives. Dr. Gilman's own task, as he informs us, "besides general supervision, has been to incorporate into the body of the work the materials prepared by Dr. Beck, and to make the changes of which he had indicated the propriety." He acknowledges his obligations to John C. Dalton, M.D., for a valuable paper on the Corpus Luteum; and to George Shea, Esq., counselor-at-law, for valuable suggestions on the purely legal portion of the subject; also to the Hon. Murray Hoffman, of the New York Superior Court, for favors of the same kind.

Thus invigorated and enlarged by external additions, as well as by interstitial supply, through the voluntary labors of able and judicious friends, this originally sterling treatise, modestly called Elements, again comes before us, as a methodically arranged repertory, complete in the number of its subjects, and fuller in the details of most of them than any kindred work extant. Like the famed river praised by the poet, it is "to overflowing full," rich to exuberance, and requiring time to turn to ready account its multifarious details. If it have a defect, it is in its voluminous character, which, while it gratifies the studious reader who has leisure on his side, is not favorable to a ready reference at the hurried moment of need, with a view of clearly ascertaining the actual principle, rule, or law, deduced from the many facts and sometimes conflicting opinions which are brought to bear in elucidating the case.

After "taking a retrospective view of the great field of medical jurisprudence, and of those who have acted a prominent part in it," and giving an analysis of the large work of M. Briand on the subject, in our November number of last year, we are not now called on to give the history of the science, to enlarge on its importance, nor to engage in a critical disquisition on the "Elements of Medical Jurisprudence." We cannot, however, forbear, even at the risk of wearying by iteration, from renewing the expression of our deep regret and mortification at the general neglect of medical jurisprudence in the larger number of the medical schools of the United States, as publicly evinced in their failure to recognize it as a distinct branch, and to teach it from a separate chair. This neglect is more complete and signal in the three great medical schools of Philadelphia than in most of the institutions of the kind in other places. In these latter acknowledgments of the utility if not paramount importance of the subject are implied by an announcement, in the curriculum, of an intention on the part of some one of the Faculty to teach it in connection with his specific subject. In the published statement of branches and professors, emanating annually from the medical schools of Philadelphia, there is not even the slightest mention of medical jurisprudence, nor any intimation given that it will engage the attention of a professor more than Hindu mythology, or the squaring of the circle. The only resource left to the students of medicine and law under such privations, is in the careful perusal of works like the one now before us; and certainly it must be admitted that the compensation is not small.

For reasons already assigned, as well as from the fact that the Elements of Medical Jurisprudence have been brought so repeatedly into public notice in reviews and notices of the successive editions which it has gone through, we shall attempt to give scarcely more than an outline of the contents of the two volumes of which the work consists, while directing, at the same time, the attention of our readers to the subjects on which additions are made in the last edition by parties previously named.

After an introduction, giving the definitions and history of medical jurisprudence, the first volume opens with a chapter on "Feigned Diseases," to which apposite additions have been made by Dr. Coolidge. The greater number of cases of this kind of imposture present themselves to army and navy medical officers, by men who are desirous of procuring their discharge. The patience and pertinacity which these malingerers evince, and their endurance of the severe and painful tests to which they are subjected, are truly wonderful. In common life, women are the most numerous and the most adroit at simulating infirmities of all kinds—in some instances for the purposes of gain, or other direct personal advantage, in others from the mere love of mystery and deception. While the most remarkable examples of feigned diseases have been met with among soldiers and sailors, and the female inmates of hospitals, and also in criminals, for the purpose of escaping the penalty of the law, the physician in his daily routine of practice should be prepared to meet, every now and then, with cases which properly come under this head, even when they do not seem to be more than common exaggerations of real infirmities. This is a kind of simulation called by Zacchias *simulatio latens.* The whole subject forms one of the many chapters of human life in which there is a keen struggle and encounter of wits between the deceiver and the detector—and on this account alone, irrespective of the design on the part of the historian to aid the cause of justice, its perusal has about it something of a romantic interest.

The principal diseases that have been feigned are fevers, affections of the heart, pulmonary consumption, hepatitis, various disorders in which pain is the predominant and most distressing symptom, hemoptysis, hematemesis, bloody urine, hemorrhoids, jaundice, paleness of the skin, cachexia and great weakness, diarrhœa and dysentery, involuntary stools, vomiting, apoplexy, vertigo and headache, paralysis, epilepsy, convulsions, chorea, catalepsy, syncope and hysteria, insensibility, somnolency, hydrophobia, neuralgia, scrofula, incontinence of urine, gonorrhœa, excretion of calculi, myopia or near-sightedness, ophthalmia, nyctalopia, deafness, stuttering and stammering, tumors and enlargements, physconia, prolapsed rectum and uterus, Barbadoes leg, hydrocele, contractions and deformity, and lameness, ulcers, wounds *fictitious* and *factitious*, pregnancy and delivery, feigned insanity. "Abstinence for a great length of time is the most frequent, as well as the most successful, of these deceptions." Dr. Coolidge, repeating the question of Dr. Copland, asks: "Wherefore should we attempt to detect this deceit?" Mental alienation is sometimes feigned, and is not a little difficult of detection. A case of this nature, detailed by Mr. Marshall, is introduced by Dr. Darwall in the English edition of Dr. Beck's Elements. A work which appeared a few years back by Dr. Hector Gavin, being a "Prize Essay on Feigned and Factitious Diseases, chiefly among Soldiers and Seamen," is recommended to notice by Dr. Coolidge. Also, "*Aide Memoire Medico-Legal de l'Officier de Santé de l'Armée de Tevre,*" par F. C. Maillot et J. A. A. Puel."

The next chapter, on "Disqualifying Diseases," has also received important additions, and been in part rewritten by Dr. Coolidge. Notice is taken of disqualifications in civil cases and in criminal cases, but the greater part of the chapter is occupied with a review of the disqualifications for military service. Some of the tables introduced here acquire an additional, although incidental, value by their indicating the frequency of certain diseases in the recruits examined. An aggregate of the men thus inspected, and of the diseases for which rejections were made, amounting for the first to 809,240, and that of rejections 185,982, during three years in France, shows that 21·64 per cent of the conscripts were pronounced unfit for military service. "It has been stated on good authority, that in the Peninsular war four out of ten recruits from the agricultural districts died in a few months, while six out of ten recruits from the manufacturing districts die in the same period." Valuable information relating to the recruiting system in the United States army is furnished by Dr. Coolidge.

Some notes have been added by Dr. Austin Flint to the chapters on "Impotence and Sterility," "Doubtful Sex," and "Rape." These subjects offer a wide scope for observation and commentary, and the introduction of cases, which is only allowable in a work strictly scientific, can hardly be made without a feeling of regret at the necessity of dwelling on the state and changes, the result of natural causes or of criminal intent, of parts properly designated in common language as "private." It is observed by Dr. Beck: "An individual accused of committing rape has been known to plead that he was physically incapacitated; while the legitimacy of children has been contested on a similar plea." The history of received opinions and of legislation on the subject, as we read it here, is curious and interesting. The author adopts the division made by Foderé into *absolute*, *curable*, and *accidental* or *temporary* impotence. The causes, as they occur in the male, are first noticed. Those of an absolute kind are principally owing to some malformation or defect in the genital organs; and these may be either natural or artificial. To this class we refer the following: an absolute want of the penis, also a scirrhous or a paralytic state of the organ, induced by injury to the nerves or muscles of the parts, and an unnatural perforation of the penis; or in other words, the extremity of the canal of the urethra terminating at some place rather than the natural situation. When this happens in the upper part, it is styled *Epispadias;* when below, the *Hypospadias.* We shall see, however, that it would be unsafe to consider all or most of these as absolute causes of impotence. The apparent want of testes, owing to these organs not having descended from the abdomen, is no proof of impotence. The same remark may be made of the loss of one testicle only.

"Among the curable causes of impotence may be enumerated the following: an atony of the parts, arising sometimes from local disease or external injury, and at others from masturbation; a retraction of the penis, originating from stone in the bladder, or some other urinary diseases; a natural phymosis, which sometimes confines the glans in such a manner as to prevent the emission of semen; obliteration of the canal of the urethra, from stricture or other causes; and lastly, the malformation of which we have spoken, as in the place of the aperture of the urethral canal. All these have been successfully obviated by modern surgery."*

---

* We do not introduce the foot-notes of this paragraph. These additions are numerous in the work, and would of themselves almost constitute a body of legal medicine.

"The third class of causes of impotence, the accidental or temporary ones, is the most important, as they are frequently the subject of legal investigation. They are those which affect an individual during his marriage, and of course have to be considered in cases of contested paternity. The law presumes that the husband is the father of every child conceived during the term of wedlock, yet it allows an investigation as to the chastity of the female." "The proofs of bastardy may be thus: 1. impotence, and 2. a proof of non-access, so conclusive that it is *impossible* that the husband could have been the father of the child."

In treating of impotence and sterility in the female, we may divide them, as in the case of those of the male, into incurable and curable. The incurable causes are: 1. An obliteration or thickening of the sexual organs, so as to prevent any introduction. 2. A natural or fistulous communication of the vagina with the bladder or rectum. There are exceptions, however, to this being an insuperable bar to conception. 3. A prolapsus or retrocession of the uterus, or a prolapsus of the vagina. "These are of course curable during their first stage." 4. A cancer of the vagina or uterus. Cases are on record of females thus diseased having borne children. 5. Extreme brevity of the vagina (congenital) would seem to be occasionally an incurable cause, so far as relates to the pain caused by connection, although it probably may not be connected with sterility. The curable cases are 1. A dense substance covering the orifice of the vagina. 2. An extreme narrowness of the vagina. 3. A similar condition caused by tumors and callosities, cicatrixes remaining after the cure of ulcers, or from lacerations after difficult labor. "A dilatation of these may be made according to the rules of modern surgery." To the above causes may be added long-continued hemorrhage, recent prolapsus of the uterus or vagina, or even protracted fluor albus. A protrusion of the bladder into the vagina is suggested by M. Ingleby as an additional cause of impotence.

The causes of sterility of an incurable nature are "a scirrhous or cartilaginous uterus, stricture in the cavity of that organ, a polypus on the interior of the uterus, enlarged and scirrhous ovaria." "The causes which may be curable are, obliquity in the position of the uterus, too great irritability of that organ, excessive menstruation, leucorrhœa, retention of the menses. This last, however, is not by any means a certain cause of sterility, as women have become pregnant without the menses ever occurring." There are many causes of sterility which we cannot explain. Ashwell ascribes it to four principal causes: too early marriage, general ill health, too frequent sexual intercourse, and dysmenorrhœa.

The next chapter, on "Doubtful Sex," contains a great amount of curious matter, in which, of course, the subject of hermaphrodites takes a prominent place. Reference is made by the author to an elaborate essay by Andrew Duncan, Jr., M.D.; also to the articles *Generation*, in

Rees' Cyclopedia, and *Hermaphrodites*, in Brewster's; several articles on the subject by Marc, in the *Dictionnaire des Sciences Médicales;* and a very learned and elaborate article on "Hermaphroditism," by Professor Simpson, in the Cyclopedia of Anatomy and Surgery, vol. xxi. The researches of modern anatomists, says a writer in the *British and Foreign Medical Review*, have completely set at rest the long-debated question of hermaphrodism in the vulgar acceptance of the word. It is anatomically and physiologically impossible. He makes the important additional remark, that legislation, admitting only two grand classes of individuals, on whom it imposes duties, and grants different and almost opposite rights, according to their sex, does not truly embrace the entire of the cases; for *there are subjects who have* really no sex, such as neuter hermaphrodites, and hermaphrodites mixed by superposition; and, on the other hand, certain individuals, the bisexual hermaphrodites, who present the two sexes united in the same degree.

"Rape," the subject of the next chapter, ever excites a deep interest, not alone in its medico-legal relations, but from a lively sympathy for a helpless, and, for the most part, young female, whose condition and age ought to be not only a protection against violence, but a claim for attention and kindness. The author begins with a notice of the signs of virginity, of which the hymen is generally regarded as one of the most constant and reliable. But before we deduce a positive opinion from the presence or absence of this membrane, we should be aware that "*it may be wanting from original malconformation, or it may be destroyed by disease or some other causes, and yet the female be pure.*" "*This membrane may, on the other hand, be present, and yet the female be unchaste; nay, she may become pregnant without having it destroyed.*" It has been found in girls who were laboring under syphilis. Narrowness of the vagina cannot be much relied on as a sign. The fleshy tubercles called *carunculæ myrtiformes*, commonly spoken of as the remains of the hymen, may, it is now positively stated, coexist with this latter. In treating of the signs of defloration and rape, the medical legist must bear in mind the fact that disease sometimes produces the appearance of external injury, and leads to suspicions against innocent persons. In some cases of typhus fever there is a mortification of the pudenda; but it is also a proof that a similar state of things may be caused by violence. "Marks of external injury are hence to be considered as *corroborating*, but not as *certain* proofs of the commission of a rape."

Doubts have been entertained whether a rape can be consummated on a grown female in good health and strength. "Metzger only allows of three cases in which the crime can be consummated: where narcotics have been administered, where many are engaged against the female, and where a strong man attacks one who has not arrived at the age of puberty." In addition to these circumstances, we must admit "that terror may operate on a helpless female; she may resist for a long time, and then faint from fatigue; or the dread of instant murder may lead to the abandonment of active resistance." A question comes up, whether "a female can be violated during sleep without her knowledge?" "As to natural sleep I totally disbelieve in its possibility with a pure person," is the decided opinion of the author. The case is different where the sleep has been caused by powerful narcotics, by intoxication, or if syncope or excessive fatigue be present. The general opinion that pregnancy cannot follow rape is contradicted by exceptional cases, although they be few in number.

The chapter on "Pregnancy" abounds with historical and physiological lore, which is made the foundation for a review of the subject in its medico-legal bearings. "Few questions occur in legal medicine of greater importance than the one we are about considering. On its proper decision may depend the property, honor, or the life of the female." Ought we not to add, also, the reputation and rights of children, or of those claiming to be such? Among the various signs of pregnancy recorded and examined in this chapter, is a *bluish tint of the vagina* extending from the os externum to the os uteri. This is regarded by Dr. Kluge, professor of midwifery in Berlin, as a sure test. An analogous one, amounting to a change of color on the lining membrane of the vagina, so as to be of a violet hue, and sometimes purplish like the lees of wine, was noticed by M. Jacquemin. Dr. Montgomery, of Dublin, acknowledges the importance of this sign. Doubts have recently been thrown over the validity of the indications furnished by auscultation, to ascertain the presence of a living fœtus in the womb. It is obviously improper to rely on any single proof of pregnancy.

We are obliged to pass over, reluctantly, the long chapter on "Delivery," concealed and pretended, its signs, and the questions of the viability of the child and its ability to inherit; and without our being able to rest on the following one, or "Infanticide." This last, by Dr. John B. Beck, is one of the completest treatises on the subject extant. It takes up more than 160 pages of the first volume, that now under notice. "Legitimacy," the subject of chapter ninth, brings up the questions of the ordinary term of gestation, premature delivery, and protracted delivery; the laws of various countries on the subject of legitimacy, and inquiries relating to paternity and filiation. "Presumptions of Survivorship" involve considerations of a mixed nature in which the medical testimony has a collateral value. "Age and Identity,"

themes for another chapter, furnish striking evidences of the fallibility of human testimony, and of a credulity which, in some instances, seems to be endemic, affecting the memory and powers of observation of the inhabitants of an entire town or district, and even of the immediate relatives of the person whose place is attempted to be taken surreptitiously by an intruding impostor. Cases of this nature yield ample materials for the "*Causes Célèbres*," which one reads with a more lively interest than would be produced by an elaborated romance. "Insurance upon Lives" is the title of a chapter, the subjects of which appeal to the pecuniary sensibility of an increasing class of persons. Of all the subjects which come within the province of medical jurisprudence there is no one which produces so pervading, and, at the same time, painful interest, and which tasks so severely the reasoning faculties of those who engage in its investigation, as insanity or mental aberration. Its very fullness, and the many doubtful points to be discussed for its elucidation, forbid any attempt on our part to give even the slightest sketch of the chapter on "Mental Alienation," which concludes the first volume of the "Elements." We notice it now, in order that we may refer to the material addition made by Dr. D. Tilden Brown, which brings up the description of the phenomena of mental diseases to more conformity with the present state of knowledge than was met with in the last edition. It was Dr. R. Beck's intention to omit entirely the chapter on this subject, with a view of his preparing, in conjunction with the late Dr. Amariah Brigham, a separate volume on mental diseases. Dr. Brown seizes on the occasion to recommend, in terms of decided but not ill-judged praise, the recent work of Drs. Bucknill and Tuke, entitled "Psychological Medicine," of which a tolerably full analysis was given in this Journal, Sept., 1858. Dr. Gilman also adds some appropriate critical commentaries to the present chapter, the slightest glance at which must convince the tyro in legal medicine what little probability there is of his being able to extemporize an opinion on any one of the many points which may come up in a court of justice, respecting the imputed insanity of an individual.

The first chapter of the second volume, chapter fourteen of the entire work, in two parts, is on "Persons found Dead." A large amount of anatomical, physiological, and surgical knowledge and acumen are called into requisition in examinations and evidence in the trial of cases coming under this head. The topics more immediately discussed are, duties of the coroner, medico-legal dissection, sudden death from natural causes, and death from violent causes. Under this last head are asphyxia, and the question whether death arises from suicide or other violence, persons found dead from cold, from hunger, from lightning, burnt to death, and death from wounds. The question to be decided is, whether the wounds are the result of suicide, accident, or homicide. Other topics, under the general head, are persons found dead from noxious exhalations, found hung, strangled, smothered, suffocated, or drowned.

The chapter on "Wounds" has received additions such as might be expected from the well-known union of learning and practical surgery possessed by its annotator, Dr. John Watson. The extensive and complicated subject of "Poisons," itself constituting, under the head of Toxicology, a distinct branch of medical jurisprudence, and which is sometimes called Legal Chemistry, takes up nearly five hundred and fifty pages of the second volume; thus equaling in size many comprehensive treatises. Modern science, especially in the garb of analytical chemistry and microscopy, has imparted a precision of detail and accuracy of deduction to toxicology beyond the advances made in the other walks of legal medicine. There are still, however, obscurities, especially in the detection of vegetable poisons—witness the late case of Palmer, which baffle the skill and acuteness of the most practiced expert, and prevent the full and general application of a now received dictum, viz., that the symptoms of poisoning, including the morbid changes of structure, though they may induce violent suspicions of the cause, are not, of themselves, by any means conclusive, without the corroborating and final proofs furnished by chemical tests, in an examination of the substance in the body or after it has been ejected. Fortunately for the ends of justice, the vegetable poisons, which are the most difficult to detect after their ingestion into the stomach and subsequent ready absorption, give rise to symptoms of a more diagnostic nature than those of the mineral kingdom, the presence of which is so much more readily ascertained in the cavities and fluids of the body. Drs. McCready and St. John have been careful not to overlay the already copious text with long additions. Those made by them are few and short, and to the point.

The last chapter, on "Medical Evidence," to which good annotations have been made by Dr. Gilman, contains excellent advice to the inexperienced witness who is summoned before a court of law. He will find that it is necessary, not only to be acquainted with the professional bearings of the question at issue, but, also, to evince a calmness and self-possession which will command the respect of all parties and protect him from the impertinences and purposed vexations to which he may be sometimes subjected by flippant counsel on a cross-examination. Dr. Gilman judiciously remarks, "that the medical witness will find it to his advantage to manifest the greatest deference to the judge; it is far better to exceed than to fall short in this matter."

